# Evaluation of the efficacy of icariin against heat stress-induced spermatogenic dysfunction in the testes of dogs

**DOI:** 10.3389/fvets.2025.1631149

**Published:** 2025-07-02

**Authors:** Baoan Li, Liuwei Xie, Mingqiang Song, Xiumin Zhang, Chuanguo Yan, Wanpeng Gao, Wenxing Wang, Yang Yang

**Affiliations:** ^1^Police Dog Technology College, Criminal Investigation Police University of China, Shenyang, China; ^2^Institute of Subtropical Agriculture, Chinese Academy of Sciences, Changsha, China; ^3^The Second Affiliated Hospital of Shenyang Medical College, Shenyang, China

**Keywords:** icariin, dog testicular heat stress, testosterone synthesis pathway, spermatogenic dysfunction, cellular apoptosis

## Abstract

**Introduction:**

This study examines the detrimental effects of high-temperature environments on canine testicular function and reproductive health, and investigates the potential of *Epimedii Folium*, particularly its active component icariin, in alleviating these effects and improving testicular function.

**Methods:**

A completely randomized single-factor design was employed, involving 24 adult male Beagle dogs (9.82 ± 0.73 kg) assigned randomly to four treatment groups, with six dogs in each group. The groups included a negative control group (“Control”), a positive control group exposed to testicular heat stress (“Model”), and two icariin-treated groups receiving daily doses of 0.5 g/kg (Icariin-L) and 1.0 g/kg (Icariin-H), respectively. All groups, except the negative control, underwent a testicular heat stress model to induce damage and assess the effects of icariin on sperm quality, testicular function, hormone levels, protein expression, and testicular histological changes.

**Results:**

Icariin supplementation improved sperm quality under heat stress, as indicated by increased total sperm count and motility, along with a reduction in sperm malformation rate (*p* < 0.01). It also restored adenosine triphosphate (ATPase) activities (Na^+^-K^+^-ATPase, Mg^2+^-ATPase, and Ca^2+^-ATPase) and serum hormone levels (*GnRH*, *LH*, and *E2*) (*p* < 0.01). Western blot analysis revealed that icariin upregulated steroidogenic proteins (*STAR*, *17βHSD*, and *CYP450*) and the tight junction protein *ZO-1* (*p* < 0.01), while downregulating the *BAX* expression (a key regulator of mitochondrial apoptosis) and enhancing the *BCL-2* expression (a major anti-apoptotic factor in the *BCL-2* family) (*p* < 0.01). Histological assessments demonstrated that icariin mitigated heat-induced damage to seminiferous tubules, epithelial thinning, and spermatogonia degeneration. Furthermore, molecular docking analysis confirmed a strong binding affinity between icariin and *17βHSD*, mediated by hydrogen bonding and hydrophobic interactions.

**Conclusion:**

These findings suggest that icariin may alleviate testicular heat stress by modulating testosterone synthesis, enhancing ATPase function, restoring blood–testis barrier integrity, and inhibiting apoptosis. The dose-dependent efficacy (1.0 g/kg > 0.5 g/kg) supports the potential of icariin as a possible therapeutic agent for improving reproductive health in dogs exposed to high-temperature environments.

## 1 Introduction

Dogs residing in high-temperature environments, particularly southern regions of China or during the intense summer months, are frequently exposed to elevated ambient temperatures, which pose a considerable risk of heat stress to their testes. This exposure poses a significant challenge to maintaining reproductive health in dogs. The testes, as the primary organ of the male reproductive system, play a crucial role in maintaining the reproductive capacity of dogs through their normal physiological functions (1, 2). However, studies have shown that high-temperature environments can cause spermatogenic dysfunction in the testes, resulting in reduced sperm quality and quantity, and ultimately affecting the reproductive performance of dogs (3). Therefore, exploring effective strategies to mitigate testicular heat stress in dogs, along with elucidation of the underlying mechanisms, holds immense theoretical and practical importance.

*Epimedii Folium* has been found to contain more than 379 compounds, among which are flavonoids, lignans, organic acids, terpenoids, dihydrophenanthrene derivatives, alkaloids, and other compounds. Flavonoid compounds, such as epimedin A, B, C, icariin, baohuoside I, icariside I, and icaritin, are acknowledged as the leading chemical and pharmacologically active components in *Epimedii Folium* (4, 5). In recent years, icariin, a compound derived from traditional Chinese medicine and the primary active ingredient in *Epimedii Folium*, has shown extensive potential in enhancing testicular function. Chen et al. reported that a dosage of 200 mg/kg of icariin significantly increased epididymal sperm count and testosterone levels in male rats by modulating the expression of specific genes, including peripheral benzodiazepine receptors (*PBR*) and steroidogenic acute regulatory protein (*STAR*) (6). Similarly, Zhao et al. found that the administration of 50 mg/kg body weight (BW) of icariin for 80 consecutive days significantly improved the reproductive performance of male dairy goats, manifesting as increased serum levels of gonadotropin-releasing hormone (*GnRH*), luteinizing hormone (*LH*), and testosterone, enhanced spermatogenesis and sperm motility, and improved testicular organ coefficients (7). Furthermore, icariin has demonstrated remarkable efficacy in ameliorating age-related declines in spermatogenic function, safeguarding the junctional function of testicular Sertoli cells, and upregulating ERα/c-fos signaling and the *PKR* pathway. Its mechanism of action is contingent upon the presence of these signaling molecules (8). Additionally, icariin exerts protective effects against testicular damage in diabetic rats by markedly improving the morphology of the seminiferous tubules, increasing the number of spermatogenic cells, and effectively inhibiting cell apoptosis (9).

While *Epimedii Folium* contains multiple bioactive compounds, icariin is its primary flavonoid component and has demonstrated significant effects in enhancing testicular function and spermatogenesis across rodent and caprine models (6, 7, 9). Given icariin’s dominant concentration and prior evidence of spermatoprotective effects, we focused on its specific mechanisms in mitigating canine testicular heat stress. Particularly under high-temperature conditions, the manner in which icariin regulates the testosterone synthesis pathway and alleviates spermatogenic dysfunction by influencing the expression of related proteins in testicular tissue warrants further elucidation. Consequently, the present study aims to investigate the therapeutic effects and underlying mechanisms of icariin in dogs with testicular heat stress. It is expected that this study will elucidate the molecular mechanisms by which icariin alleviates heat stress in canine testes and provide a scientific basis for the application of icariin in protecting the reproductive health of dogs.

## 2 Materials and methods

All experimental procedures involving Beagle dogs were approved by the Animal Care and Welfare Committee of the Chinese Academy of Sciences (Ethical Approval No. ISA-2024-0031). The study strictly adhered to institutional guidelines for the humane use of animals, and all participating institutions obtained informed consent for the experimental protocols.

### 2.1 Experimental materials

This study utilized icariin standard reagent (B9520, purity ≥ 98%, analytical reagent (AR) grade) obtained from Meilun Biotech Co., Ltd. (MB2189-S, Dalian, Liaoning, China). An ultramicro-ATPase kit was procured from Enzyme-Linked Biotechnology Co., Ltd. (ml093096, Wuhan, Hubei, China). Additionally, the *GnRH* kit (E-EL-0071), *LH* kit (E-EL-M3053), and *E2* kit (E-OSEL-C0001) were sourced from Elabscience Biotechnology Co., Ltd. (Wuhan, Hubei, China). For antibody-based analyses, the following antibodies were purchased from Thermo Fisher Scientific: *ZO-1* (33–9100), *BAX* (MA5-14003), *BCL-2* (13–8800), *STAR* (MA5-47013), 17β-hydroxysteroid dehydrogenase (*17βHSD*) (MA5-25937), and *CYCP450* (PA1-343).

### 2.2 Experimental design and diet composition

**Figure 1:**
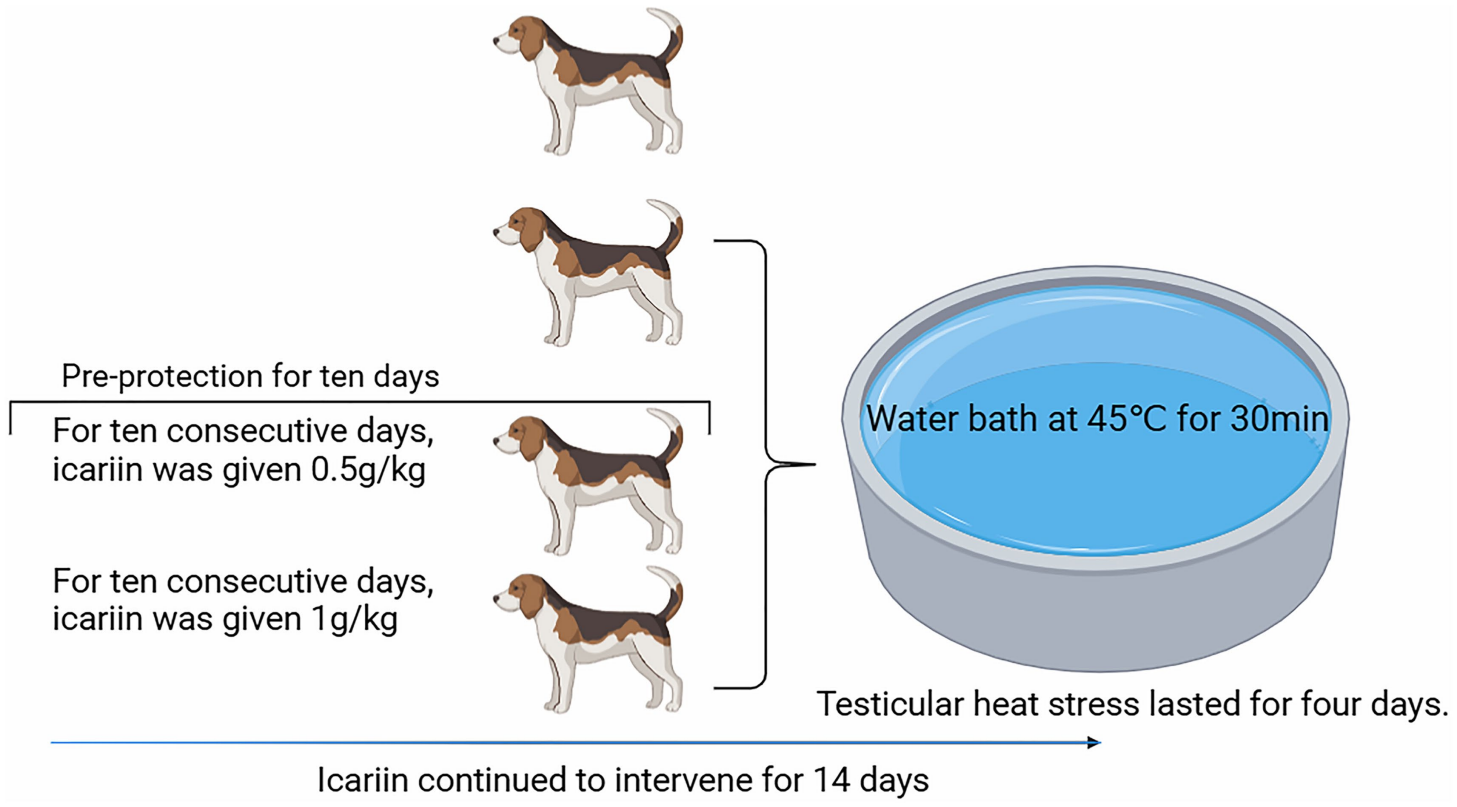
Flowchart of procedural operations for each treatment group during the formal experimental period.

This study employed a single-factor completely randomized design, utilizing 24 2-year-old adult male Beagle dogs procured from Changchun Fenglong Biotechnology Co., Ltd.—a nationally certified laboratory animal provider (Unified Social Credit Code: 91220122MACQ0YWB6G). The animals, with a mean body weight of 9.82 ± 0.73 kg, were randomly allocated into four treatment groups, each consisting of six replicates with one dog per replicate. The treatments included the following: (1) a negative control group (“Control” group), (2) a positive control group (“Model” group), (3) a group receiving a daily dose of 0.5 g/kg icariin (icariin-L group), and (4) a group receiving a daily dose of 1.0 g/kg icariin (Icariin-H group). The diet formulation and nutrient composition, presented in Table 1, met the nutrient requirements for dogs, as outlined by the National Research Council (NRC) (10). All groups received the same basal diet, with icariin supplementation only in the treatment groups. The experimental period spanned 21 days, consisting of a 7-day pre-feeding phase and a 14-day main experimental phase. On day 18, a testicular heat stress model was established by exposing dogs in the Model, Icariin-L, and Icariin-H groups to a 45°C water bath for approximately 30 min/day, for 4 consecutive days, following the methodology described by Henning et al. (11). The experimental procedure is illustrated in Figure 1.

**Table 1:** Composition of the basal experimental diet (air-dry basis).

Items	Content, %
Brewers rice	21.20
Corn	21.40
Wheat	21.20
Chicken by-product meal	21.40
Chicken fat	5.34
Beet pulp	4.00
Corn gluten meal	2.39
Calcium carbonate	0.75
Potassium chloride	0.49
Salt	0.46
Dicalcium phosphate	0.87
premix1	0.50
Total, %	100.00
Nutrient content 2, %	
DM	93.90
CP	21.70
EE	14.10
TDF	4.73
CF	0.68
Starch	46.90
Ash	7.24

^1^Supplied per kilogram of premix: VE 1450 IU, VA 227000 IU, VD_3_ 27,800 IU, VB_1_ 122.5 mg, VB_2_ 262.5 mg, VB_3_ 850 mg, VB_4_ 90 mg, VB_6_ 75 mg, VB_12_ 1.85 mg, Fe 1,600 mg, Cu 330 mg, Zn 3,000 mg, Mn 230 mg, I 47 mg, Se 17.5 mg, Ca 19,000 mg, Na 35,000 mg, Cl 60,000 mg, and Mg 30,000 mg.

^2^Nutrient content was measured values (air-dry basis).

### 2.3 Housing management and sample collection procedures

Prior to the experiment, dogs were housed individually and underwent standard procedures, including vaccination, deworming, and labeling (1–24). During the pre-feeding phase, all dogs were fed the same diet. In the main experiment phase, dogs were fed their respective treatment diets. Each dog received 600 g of diet per day, divided into two equal feedings (morning and evening), with *ad libitum* access to water. Dogs were allowed at least 60 min of free activity, which started 40 min after each feeding. The experimental facility was regularly cleaned and disinfected throughout the study duration. On day 22, venous blood was collected from all dogs. Five milliliters of blood were placed in a coagulation-promoting tube, allowed to stand for 30 min, and subsequently centrifuged at 2,500 rpm for 10 min. The serum was separated and stored at −20°C. All surgical interventions (orchiectomy) were performed under general anesthesia using isoflurane inhalation (3% induction, 2% maintenance). Pre- operative and postoperative analgesia was administered via subcutaneous buprenorphine (0.02 mg/kg) and oral meloxicam (0.1 mg/kg). No animals were euthanized, and all dogs fully recovered post-surgery under veterinary supervision.

### 2.4 Sperm quality, testicular function, and hormone level determination

After testicle removal, the epididymal portion was excised and rinsed with PBS buffer to obtain spermatozoa. The collected spermatozoa were stained and then analyzed using a sperm motility analyzer (HT-Motility™, Hamilton Thorne, Inc, Beverly, MA) to assess their density, viability, and abnormality rate. The testicular tissue was homogenized, and the activities of Na^+^-K^+^-ATPase, Mg^2+^-ATPase, and Ca^2+^-ATPase were measured using standard kit procedures. The collected serum was assayed for *GnRH*, *LH*, and *E2* levels using the respective standard kits.

### 2.5 Testicular histopathology and protein expression analysis

Testicular tissue specimens (0.5 cm^3^) from dogs were fixed by immersion in 4% paraformaldehyde (PFA, pH 7.4) at 4°C for 48 h, followed by graded ethanol dehydration and xylene clearing. The processed tissues were embedded in paraffin and sectioned at a thickness of 5 μm using a rotary microtome (RM2235, Leica Microsystems). Sections were stained with hematoxylin and eosin (H&E) according to standard protocols and then mounted with neutral balsam for histomorphological examination under a light microscope (DM4000B, Leica Microsystems). For Western blot analysis, parallel samples were homogenized in ice-cold RIPA lysis buffer (Catalog No. R0020, Solarbio Life Sciences) containing a 1% protease inhibitor cocktail (Catalog No. 04693132001, Roche Diagnostics) using a Polytron homogenizer. Lysates were centrifuged at 14,000 *g* for 15 min at 4°C, and the supernatants were collected for protein quantification via BCA assay (Catalog No. 23225, Pierce™, Thermo Fisher Scientific) with bovine serum albumin standards. Protein extracts (30 μg per lane) were separated on 12% sodium dodecyl sulfate (SDS)-polyacrylamide gels and transferred to activated polyvinylidene fluoride (PVDF) membranes (0.22 μm, Catalog No. IPVH00010, MilliporeSigma) using a wet transfer system. Membranes were blocked with 5% bovine serum albumin (BSA) (w/v) in tris-buffered saline (TBST) with 0.1% Tween-20 for 2 h at room temperature and then incubated with primary antibodies at 4°C for 16 h with gentle agitation: *ZO-1* (1:1,000), *BAX* (1:1,000), *BCL-2* (1:1,000), *STAR* (1:800), *17βHSD* (1:1,000), and *CYP450* (1:1,000). After five 5-min TBST washes, membranes were incubated with horseradish peroxidase (HRP)-conjugated goat anti-rabbit immunoglobulin G (IgG) secondary antibody (1:10,000) for 1 h at 25°C. Protein bands were visualized using SuperSignal™ West Femto Chemiluminescent Substrate (Catalog No. 34095, Thermo Fisher Scientific) and captured using a FUSION FX Spectra imaging system (Vilber Lourmat). Densitometric analysis was performed using ImageJ 1.53 k with β-actin (1:5000, Catalog No. AC004, ABclonal, Wuhan, Hubei, China) as the loading control.

### 2.6 Molecular docking and visualization analysis

Small molecule ligand two-dimensional (2D) structures were retrieved from the PubChem CID database (http://pubchem.ncbi.nlm.nih.gov/), converted into three-dimensional (3D) structures using ChemOffice 20.0 software (PerkinElmer, Inc., Waltham, MA), and subsequently saved as mol2 files. High-resolution crystal structures of protein targets were selected from the Research Collaboratory for Structural Bioinformatics – Protein Data Bank (RCSB PDB) database (http://www.rcsb.org/) to serve as molecular docking receptors. PyMOL 2.6.0 software was employed to remove water molecules and phosphates from the protein, add hydrogen atoms to the ligand and the protein, and prepare them for docking. These were saved as Protein Data Bank identification (PDB ID) files. The Molecular Operating Environment (MOE, Dassault Systèmes BIOVIA, San Diego, CA) 2019 software was used to minimize the energy of compounds, preprocess target proteins, and identify active sites. MOE 2019 was chosen for docking, and docking was performed with 50 iterations to ensure thorough exploration of the binding space. The binding activity was evaluated based on binding energy, and the results were visualized using PyMOL 2.6.0 and Discovery Studio 2019 software (Schrödinger, LLC, New York, NY).

### 2.7 Statistical analysis

All data are presented as mean ± standard deviation (SD), and fundamental statistical analyses were performed using the MEANS module of the Statistical Analysis System (SAS Institute Inc. Cary, NC) 9.4. The Generalized Linear Model (GLM) module was employed for the analysis of variance (ANOVA) of sperm quality, testicular function, hormone levels, and expression of heat stress-related proteins among different treatments. Duncan’s multiple comparison test was used to assess the effect of icariin on the ability of canine testes to resist heat stress-induced damage. A significance level of *p* of < 0.05 was considered statistically significant, and a *p*-value of < 0.01 indicated a highly significant difference. Statistical graphs were generated using GraphPad Prism 8.0 (GraphPad Software, LLC, San Diego, CA).

## 3 Results

### 3.1 Impact of icariin on canine sperm quality

**Figure 2:**
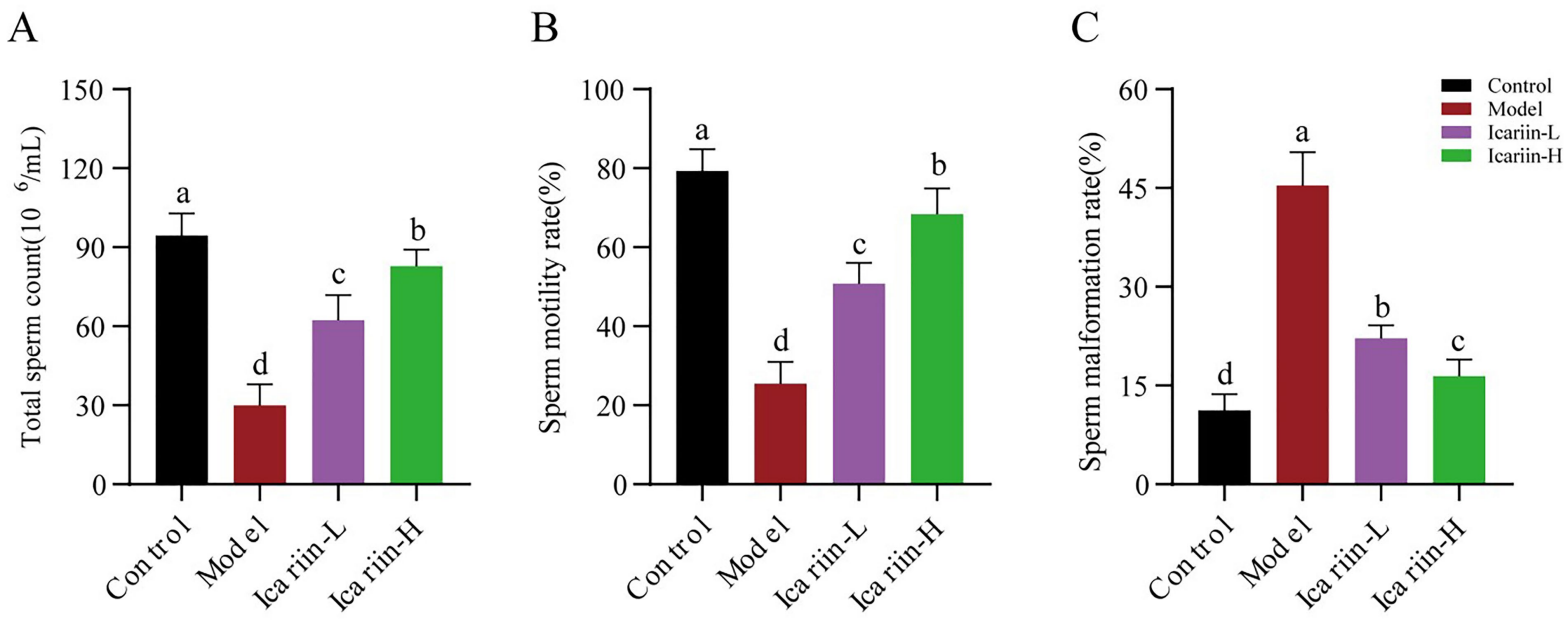
Effects of icariin on canine sperm quality under heat stress. **(A)**, Total sperm count; **(B)** Sperm motility rate; **(C)** Sperm malformation rate. Letters labeled differently represent significant differences, *p* < 0.05.

As shown in Figure 2, the heat-stressed dogs (Model group) exhibited a significant decline in total sperm count and motility compared with the Control group (*p* < 0.01) and were accompanied by a marked increase in the sperm abnormality rate (*p* < 0.01). Dietary supplementation with icariin has led to significant improvements in these parameters. Specifically, compared with the Model group, both Icariin-L and Icariin-H groups demonstrated a significant increase in total sperm count and sperm motility rate (*p* < 0.01), while also significantly reducing sperm malformation rate (*p* < 0.01). Furthermore, high-dose icariin addition demonstrated superior efficacy in canine sperm quality (*p* < 0.01).

### 3.2 Impact of icariin on reproductive function status

**Figure 3:**
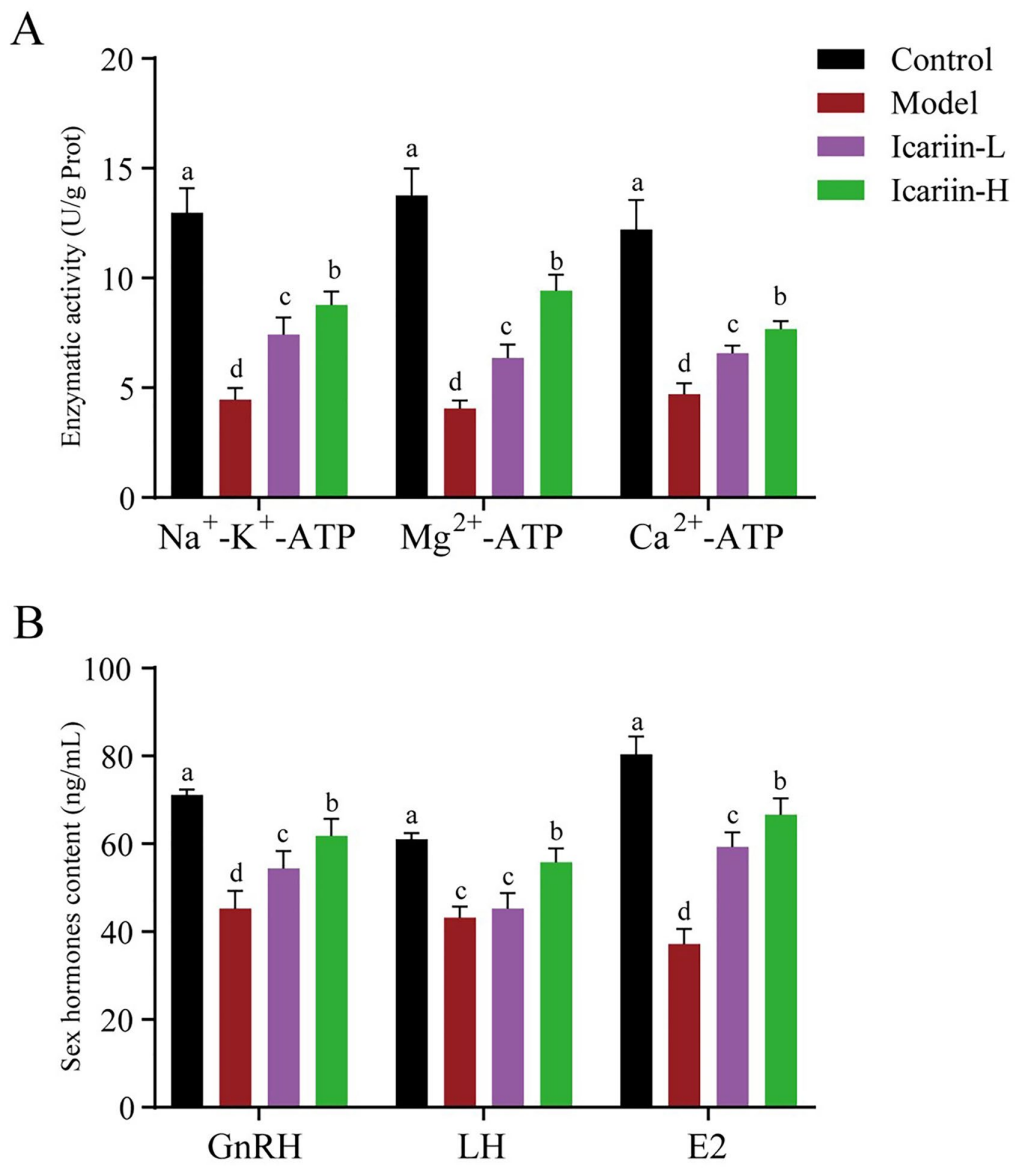
Impact of icariin on testicular ATPase activity and serum hormone levels. **(A)** Enzymatic activity; **(B)** Sex hormones content. Letters labeled differently represent significant differences, *p* < 0.05.

The activities of P-type ATPases (Na^+^-K^+^-ATP, Mg^2+^-ATP, and Ca^2+^-ATP) of testicular tissue are presented in Figure 3. According to the findings, heat stress treatment significantly reduced the activity of Na^+^-K^+^-ATP, Mg^2+^-ATP, and Ca^2+^-ATP enzymes in test dogs (*p* < 0.01). In the icariin-supplemented groups (Icariin-L and Icariin-H), there was a significant improvement in the activities of Na^+^-K^+^-ATP, Mg^2+^-ATP, and Ca^2+^-ATP enzymes (*p* < 0.01). Nevertheless, compared to the Control group, the activity of P-type ATPases was still significantly reduced (*p* < 0.01). In terms of serum sex hormones, the contents of *GnRH*, *LH*, and *E2* were decreased considerably in the Model group (*p* < 0.01). In contrast, both Icariin-L and Icariin-H groups exhibited partial restoration of hormone contents (*p* < 0.01). Notably, the high-dose icariin supplement had a more pronounced impact on restoring hormone contents (*p* < 0.01).

### 3.3 Modulation of protein expression related to canine testicular heat stress resistance by icariin

**Figure 4:**
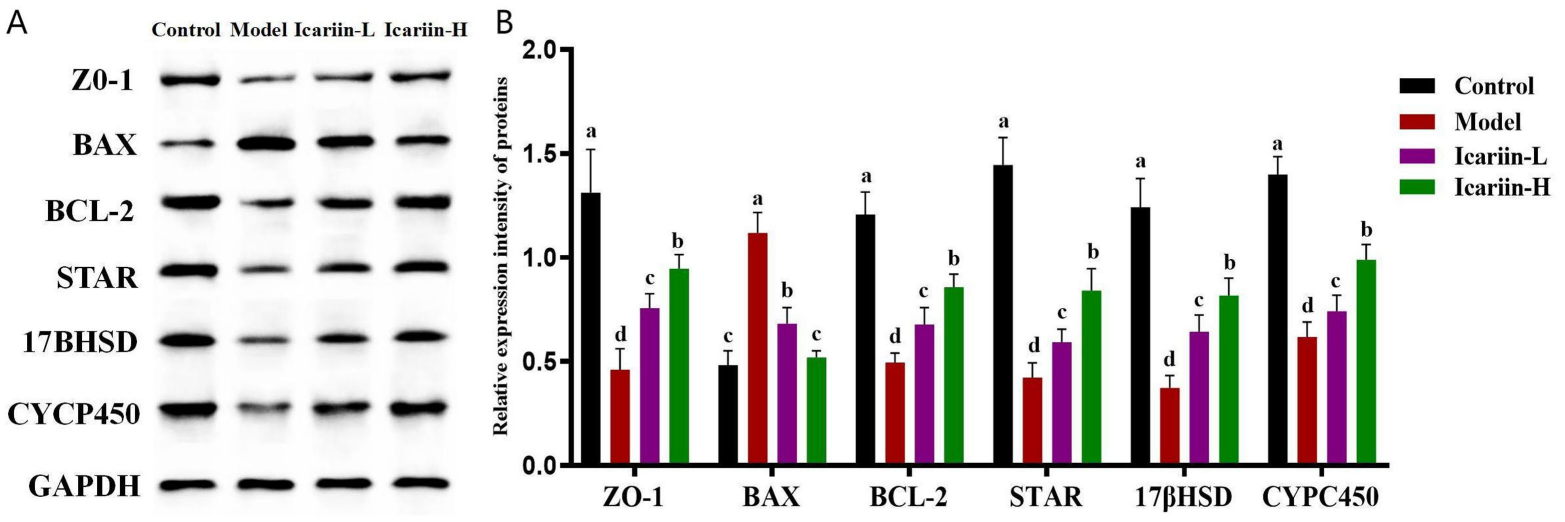
Effects of icariin on the modulation of protein expression related to canine resistance to testicular heat stress. **(A,B)** western blot analysis of *ZO-1*, *BAX*, *BCL-2*, *STAR*, *17βHSD*, *CYCP450*, and *GAPDH*. Letters labeled differently represent significant differences, *p* < 0.05.

As shown in Figure 4, the expression of *STRA*, *17βHSD*, and *CYP450* in heat-stressed dogs (Model group) was significantly decreased compared to the Control group (*p* < 0.01). Nevertheless, icariin administration significantly upregulated the expression of these rate-limiting enzymes in both the Icariin-L and Icariin-H groups, with expression levels considerably higher than those in the Model group (*p* < 0.01). Moreover, a significant reduction in *ZO-1* protein expression was observed in the testicular tissue of the Model group compared to the Control group. Notably, icariin intervention significantly increased *ZO-1* protein expression in both the Icariin-L and Icariin-H groups compared to the Model group (*p* < 0.01). With regard to testicular cell apoptosis, the Model group demonstrated a significant increase in *BAX* protein expression and a corresponding reduction in *BCL-2* protein expression compared to the Control group (*p* < 0.01). However, dietary supplemented with icariin suppressed BAX expression, a key regulator of mitochondrial apoptosis, and enhanced BCL-2 expression, a major anti-apoptotic factor in the BCL-2 family, compared to the Model group (*p* < 0.01). Furthermore, the Icariin-H group exhibited significantly greater efficacy than the Icariin-L group in enhancing resistance to testicular heat stress among dogs and modulating the expression of relevant rate-limiting enzymes and apoptotic proteins (*p* < 0.05).

### 3.4 Influence of icariin on canine testicular tissue structure

**Figure 5:**
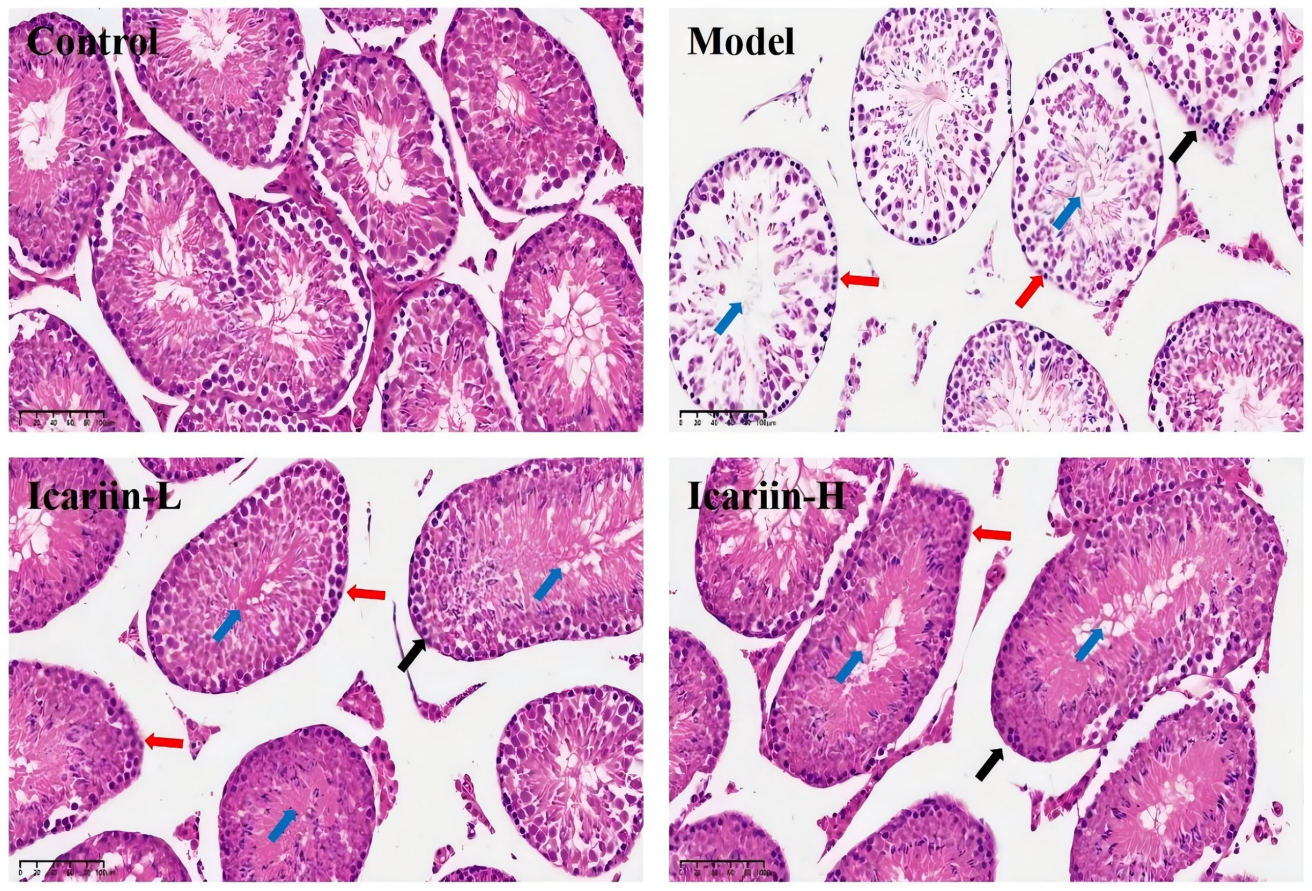
Histopathological effects of icariin on canine testicular tissue under heat stress. Representative hematoxylin and eosin (H&E)-stained sections of testicular tissue from the Control, Model, Icariin-L (0.5 g/kg), and Icariin-H (1.0 g/kg) groups. In the Model group, heat stress-induced disorganization of the seminiferous tubule (blue arrow), epithelial thinning (black arrow), and spermatogonia degeneration of spermatogonia (red arrows). icariin supplementation mitigated these lesions, with the Icariin-H group showing improved tubular architecture and reduced cellular damage. Scale bars: 100 μm.

The seminiferous tubules and testicular epithelium constitute essential elements of the testicular physiological architecture. The testicular epithelium of dogs in the Model group exhibited a significant decrease in thickness compared to the Control group (Figure 5). This reduction was accompanied by a disruption in the epithelial structure of the seminiferous tubules, as well as pronounced degeneration, detachment, and a marked decrease in the number of spermatogonia. Following dietary supplementation with icariin, notable improvements were observed in the testicular epithelium, structural integrity of the seminiferous tubule epithelium, and spermatogonia count in the Icariin-L and Icariin-H groups compared to the Model group. Notably, a discernible disparity remains in the restoration of testicular tissue connectivity and the resolution of surrounding fragmented tissue within the seminiferous tubules in these two treatment groups, as compared to the Control group.

### 3.5 Molecular docking analysis of icariin with the *17βHSD* protein target

**Figure 6:**
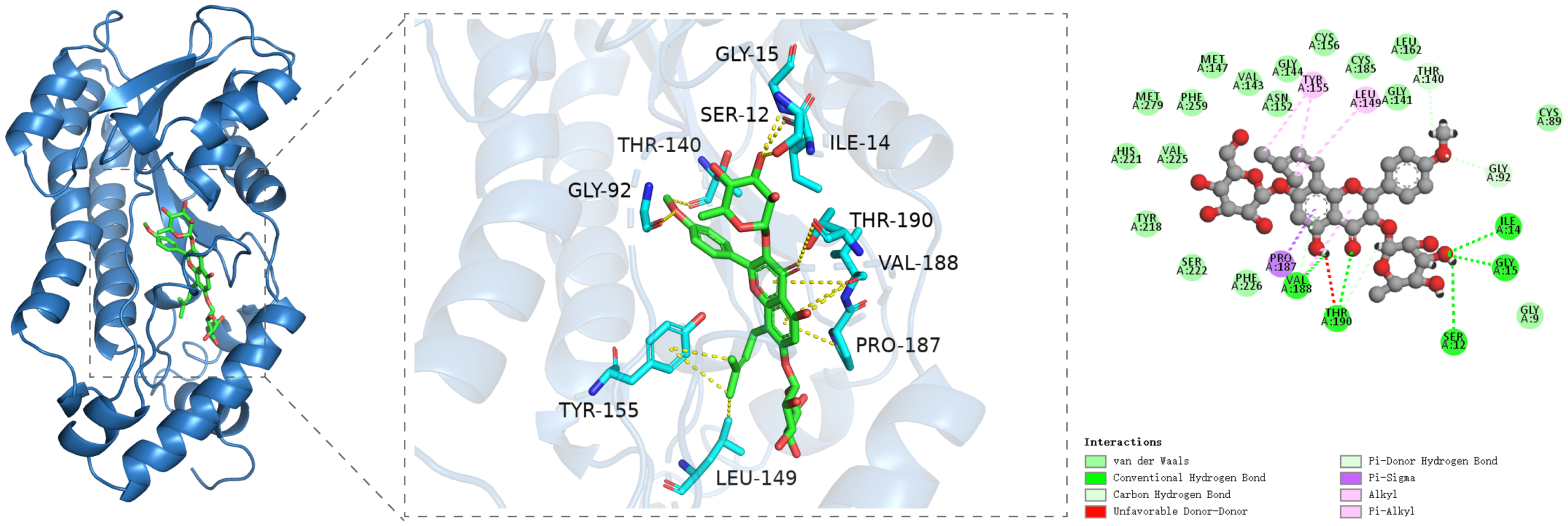
Molecular docking analysis of icariin with *17βHSD* reveals binding interactions critical for testosterone synthesis. **(A)** Three-dimensional (3D) structure of the *17βHSD* protein (ribbon diagram). **(B)** Direct interaction sites: icariin forms hydrogen bonds with Ile34, Gly15, Ser12, Thr190, and Val188 (dashed lines) and carbon-hydrogen bonds with Thr140, Gly92, and Val188. **(C)** Hydrophobic interactions (van der Waals forces) between icariin and Tyr155, Leu149, Val188, and Pro187 (Pi-Sigma interaction).

The molecular docking outcomes of icariin with the *17βHSD* protein target are shown in Figure 6. Icariin effectively penetrates the binding pocket of the *17βHSD* protein, engaging in hydrogen bonding interactions with the Ile34, Gly15, Ser12, Thr190, and Val188 residues of the protein receptor. Moreover, icariin exhibits carbon-hydrogen interactions with the Thr140, Gly92, and Val188 residues, with Thr190 notably participating in multiple interaction types. A specific Pi-Sigma interaction is observed between the Pro187 residue and icariin. Additionally, significant hydrophobic interactions are established between icariin and the Tyr155, Leu149, and Val188 residues of the protein receptor, with Val188 playing a pivotal role in multiple interaction networks.

## 4 Discussion

In mammals, prolonged exposure to hyperthermic environments disrupts systemic homeostasis by exceeding physiological thermoregulatory thresholds (12). The testes, which require a temperature of 2 to 8°C below core body temperature for normal spermatogenesis, are particularly vulnerable in dogs due to compromised scrotal cooling mechanisms (13, 14). To model this pathology, we subjected dogs to scrotal immersion in a 45°C water bath for 30 min, a protocol adapted from Henning et al. (11) that minimizes confounding stressors through non-invasive thermal exposure. Histopathological analyses revealed seminiferous epithelial thinning, disorganization of spermatogenic cell layers, and sloughing of spermatogonia in heat-stressed testes, concomitant with significant declines in sperm concentration (a 68.4% reduction compared to the control) and progressive motility (67.9% reduction). These morphological and functional impairments correlated with suppressed serum testosterone levels, suggesting partial mediation through the androgen signaling pathways. The observed reproductive dysfunction aligns with previous reports of heat-induced testicular atrophy and steroidogenic suppression in mammals (15). Plant saponins, including icariin, have shown promise in ameliorating high-temperature-induced spermatogenesis disorders in various animal models (14, 16, 17). Icariin, in particular, has demonstrated significant effects in regulating testosterone synthesis, protecting testicular tissue structure, and inhibiting cell apoptosis (6, 7, 9). Therefore, we selected icariin as the intervention in this study and, based on the metabolic body weight of the experimental animals (18), set the dietary icariin supplementation level between 0 and 1 g/kg to systematically evaluate its effects on canine sperm quality, testicular function, hormone levels, related protein expression, and testicular tissue structure.

Thermal stress induces mitochondrial oxidative damage through reactive oxygen species (*ROS*) overproduction, particularly superoxide anion (O₂^−^) and hydroxyl radical (OH^−^), which preferentially target electron transport chain complexes (19–21). This oxidative insult disrupts ATP synthesis, leading to substrate limitation for ATP-dependent membrane transporters. Specifically, Na^+^-K^+^-ATPase and Ca^2+^-ATPase activities in testicular tissue decreased by 65.6 and 61.5%, respectively, in heat-stressed dogs. Given the critical role of these enzymes in maintaining membrane potential and calcium homeostasis, their functional impairment is likely to contribute to the observed deficits in sperm motility. Notably, icariin supplementation restored testicular ATPase activities in a dose-dependent manner (22.9 and 15.4% recovery at 0.5 g/kg; 33.2 and 24.3% at 1 g/kg for Na^+^-K^+^-ATPase and Ca^2+^-ATPase, respectively), paralleling improvements in sperm motility parameters. The dose-dependent efficacy of icariin (1.0 g/kg > 0.5 g/kg) aligns with its role in modulating testosterone synthesis and cellular resilience. Higher doses more robustly enhanced ATPase activity, restored blood–testis barrier integrity via *ZO-1* upregulation, and suppressed apoptosis (reduced *BAX/BCL-2* ratio), collectively explaining superior spermatogenic recovery in the Icariin-H group. These data suggest that icariin mitigates oxidative damage through ion transport modulation, potentially via direct ROS scavenging or upregulation of antioxidant enzymes, as demonstrated in murine models (22).

Hypothalamic–pituitary–gonadal (HPG) axis dysregulation has emerged as a key mechanism underlying heat-induced reproductive failure (23). Nevertheless, this axis can be perturbed by external stimuli, such as inflammation and oxidative stress, which can impair spermatogenesis. Our data corroborate previous reports of *HPG* axis dysregulation under thermal stress. Specifically, heat-exposed dogs exhibited suppressed hypothalamic *GnRH* release, resulting in decreased pituitary *LH* production and subsequent reductions in *E2* synthesis. Post-treatment, serum *GnRH* and *LH* levels recovered to baseline values, while *E2* levels showed a gradual increase. Heat stress activates the hypothalamic–pituitary–adrenal axis, leading to elevated cortisol levels. Cortisol exerts a negative feedback effect on hypothalamic *GnRH* synthesis, thereby reducing the synthesis of *GnRH*. Since *GnRH* is critical for pituitary *LH* production, this suppression consequently diminishes LH synthesis. The restoration of *HPG* axis function coincided with improved blood–testis barrier (BTB) integrity. Immunoblotting revealed that *ZO-1* expression increased by 2.1-fold, while the *BAX/BCL-2* ratio decreased from 2.4 to 0.6, suggesting dual mechanisms of junctional stabilization and apoptosis suppression. The restoration of these proteins’ expression levels is likely instrumental in the recovery of testicular physiological function (23–25). Moreover, our study revealed that, under heat stress conditions, the testes of the Model group exhibited considerable structural damage, manifested by the emergence of large gaps and morphological distortions in the seminiferous tubules. Western blot analysis validated that the expression levels of *ZO-1* and *BCL-2* were substantially decreased, while the level of *BAX* was significantly increased in the testicular tissue of the experimental dogs. Notably, following icariin intervention, the expression levels of *ZO-1* and *BCL-2* recovered to varying extents, suggesting a potential restoration of the BTB structure. These findings imply that icariin not only modulates testosterone secretion but also effectively ameliorates testicular heat stress injury by rejuvenating the integrity of the BTB (26). Speculatively, icariin may activate *Nrf2/HO-1* signaling or directly scavenge hydroxyl radicals, as its flavonoid structure contains phenolic hydroxyl groups capable of donating protons to neutralize *ROS*. These mechanisms could synergize with ion transport regulation to reduce oxidative injury to spermatogenic cells. Notably, *BAX* and *BCL-2* extend beyond apoptotic regulation. *BCL-2* family proteins also modulate autophagy via interactions with Beclin-1, and *BAX* has been associated with mitochondrial dynamics and cellular metabolism (27, 28).

The maintenance of a stable testosterone concentration within the seminiferous tubules is crucial for the differentiation of spermatogonia into mature spermatozoa (29–31). Heat stress significantly suppressed key steroidogenic enzymes (*STAR*: 70.7% reduction; *17βHSD*: 69.9%; *CYP450*: 55.8%), with these reductions mirroring concomitant declines in testosterone levels, a pattern consistent with the findings from Ji et al. (2024) (32). Molecular docking studies revealed a strong binding affinity between icariin and *17βHSD*, consistent with the observed upregulation of steroidogenic proteins post-treatment. Concomitant increases in *STAR* and *CYP450* expression (key regulators of testosterone biosynthesis) suggest a coordinated enhancement of the steroidogenic pathway, where icariin’s binding to *17βHSD* serves as a mechanistic trigger. These findings align with the restoration of serum testosterone levels (implied by *GnRH/LH* recovery) and testicular ATPase activities, collectively indicating that icariin modulates both enzyme structure and pathway dynamics to mitigate heat stress–induced steroidogenic dysfunction. Importantly, the high-dose icariin group (1 g/kg) exhibited complete restoration of sperm count (82.9 × 10^6^ vs. 94.5 × 10^6^/mL in controls). This finding was further corroborated by Western blot analysis, which showed a significant increase in the expression levels of *STAR*, *17βHSD*, and *CYP450* following icariin treatment. These results suggest that icariin may have a direct modulatory effect on the expression of these key proteins involved in testosterone biosynthesis. These findings align with murine studies demonstrating icariin’s safety profile at 200 mg/kg (6), while extending dose–response evidence to dogs. In the current study, we administered a higher dose of icariin (Icariin-H group: 1 g/kg) to experimental dogs, which was well within their maximum tolerable dose. Our therapeutic findings indicate that the effectiveness of icariin in mitigating heat stress-induced damage to the reproductive system increased with higher dosages. This dose-dependent response suggests that icariin is not only efficacious in protecting the reproductive system from heat stress-induced damage but also safe and devoid of side effects within the normal dosage range for dogs (33). Collectively, these preclinical data support further investigation of icariin as a therapeutic candidate for mitigating heat stress-induced reproductive dysfunction in dogs. The observed dose-dependent efficacy (up to 1 g/kg) and absence of adverse effects within the tested range warrant translational studies to establish clinical safety and optimal dosing regimens.

## 5 Conclusion

The study provides preliminary evidence suggesting that icariin may exert a multifaceted protective effect against heat stress-induced damage in canine testes. Specifically, our data indicate that it upregulates the expression of proteins crucial for testosterone synthesis, enhances ATPase activity, regulates hormone levels, promotes repair of the blood–testis barrier, and inhibits cell apoptosis. These findings contribute to the scientific basis for exploring the use of icariin in preserving canine reproductive health and highlight its potential therapeutic relevance for managing reproductive disorders associated with environmental heat stress.

## Data Availability

The original contributions presented in the study are included in the article/supplementary material, further inquiries can be directed to the corresponding author.
